# Delayed diagnosis in children with congenital heart disease: a mixed-method study

**DOI:** 10.1186/s12887-021-02667-3

**Published:** 2021-04-21

**Authors:** Indah K. Murni, Muhammad Taufik Wirawan, Linda Patmasari, Esta R. Sativa, Nadya Arafuri, Sasmito Nugroho, Noormanto

**Affiliations:** Department of Child Health, Dr. Sardjito Hospital / Faculty of Medicine, Public Health and Nursing, Universitas Gadjah Mada, Jalan Kesehatan No 1, Sekip, Yogyakarta, 55281 Indonesia

**Keywords:** Delayed diagnosis, Children, Congenital heart disease, Indonesia

## Abstract

**Background:**

Delayed diagnosis of congenital heart disease (CHD) causes significant morbidity and mortality. We aimed to determine the proportion of delayed diagnosis of CHD and factors related to the delayed diagnosis.

**Methods:**

A prospective cohort study with mixed-methods was conducted in Dr. Sardjito Hospital, Yogyakarta, Indonesia. Patients aged < 18 years with newly diagnosed CHD and echocardiography confirmed CHD were included. Data were recorded from medical records and interviews from direct caregivers. Logistic regression was used to identify independent factors associated with the delay.

**Results:**

A total of 838 patients were included with median age of 2.9 years (0–17.7 years), with female predominance (54.2%, *n* = 454). The proportions of delayed diagnosis were 60.8% (510), 54.9% (373) and 86.2% (137) in all children with CHD, acyanotic and cyanotic CHD, respectively. Delayed diagnosis by doctor was the most common cause, followed by delayed diagnosis related to midwifery care, financial, referral/follow-up, and social factors. In multivariate analysis, cyanotic CHD, residence outside the city, non-syndromic, low family income, normal labour and at term gestation at birth were independently associated with the delay. At diagnosis, heart failure and pulmonary hypertension occurred in 414 (49.4%) and 132 (15.8%) children with CHD, respectively.

**Conclusions:**

Six in ten children with CHD were diagnosed with significant delay. Delayed diagnosis by doctor was the most common cause. Children with cyanotic CHD, residence outside the city, non-syndromic, low family income, normal labour and at term gestation at birth were independently associated with the delay. Comorbid complications in delayed diagnosis of CHD were prevalent.

## Background

Congenital heart disease (CHD) is the most common congenital malformation worldwide. Approximately, 1 in every 100 babies are born with CHD, with 1 in 4 births with critical CHD [1]. In Indonesia, 5 million infants are born annually [2], with approximately 50,000 infants are born with CHD, and 12,500 born with critical CHD [1]. Globally, the annual mortality rate of CHD among children has declined [3]. Despite the better survival and quality of life of children with CHD, these defects still represent a major health problem worldwide [1].

Delayed diagnosis of CHD causes significant morbidity and mortality [4]. Delayed diagnosis of CHD is associated with cardiovascular compromise and organ dysfunction leading to prolonged ventilation and mortality among neonates undergoing cardiac surgery [5].

Proper diagnosis of CHD is defined when the patient does not need emergency management at the onset of diagnosis, when treatment does not carry high risk, when there is no need for different management, or when the patient has better outcome if treated earlier. Delayed diagnosis of cyanotic CHD is when children with CHD are diagnosed after sent home from the birth clinic or hospital. Concerning acyanotic CHD, delayed diagnosis is defined when the children were diagnosed when cardiac surgery or intervention should have already been performed [6, 7].

Delayed diagnosis in congenital heart disease is prevalent globally both in high-income and low- and middle-income countries. One study in a high-income country revealed the proportion of delayed diagnosis was 8.9% including in cyanotic CHD of 10.4% and acyanotic CHD of 8.7% [7]. Another study revealed the delayed diagnosis in critical CHD is 29.5% [8]. Types of critical CHD and the presence of extracardiac defect are associated with less likely delayed diagnosis [8]. One study in a low- and middle-income country demonstrated that delayed diagnosis in congenital heart disease is 85.1% [9]. Factors contributing to delayed diagnosis in CHD are inadequately trained health system and socioeconomic constraints among those in low- and middle-income country setting [9].

Data in Indonesia concerning children with delayed diagnosis of CHD are limited. Given the existing burden of CHD in low- and middle-income countries, it is necessary to identify the magnitude of delayed diagnosis and factors associated with the delayed diagnoses in children with CHD. Accordingly, this study aimed to determine the proportion of delayed diagnosis of CHD in children and factors associated with the delay in Yogyakarta, Indonesia.

## Methods

A prospective cohort study using mixed methods with quantitative and qualitative data was conducted at the Pediatrics Department of Dr. Sardjito Hospital in Yogyakarta, Indonesia. Patients aged < 18 years old with newly diagnosed CHD between 1st February 2016 to 31st July 2017, and echocardiography-confirmed CHD were included in the study. Defects were classified into cyanotic or acyanotic CHD. Data were collected from medical records.

Patients with delayed diagnosis in cyanotic CHD were defined as newborns discharged from their birth clinic or hospital without a CHD diagnosis. For acyanotic CHD, a delayed diagnosis was defined as patients with acyanotic CHD diagnosed at an age where elective cardiac repair should have already been performed or in case immediate treatment was indicated because of the patient’s hemodynamic status [6, 7]. The expected date of cardiac surgery or intervention for congenital heart disease in children was based on the Indian guidelines for indications and timing of intervention for common congenital heart diseases [10].

The reasons associated with delayed diagnosis of CHD were obtained through interviews using open-ended questions from direct caregivers including patient’s mother, father or guardian. The caregivers were interviewed to describe their background and identify the various factors associated with delayed diagnosis such as delayed diagnosis by doctors, routine midwifery care, delayed referral/follow-up, social or financial factors. The interviews were conducted directly at the onset of diagnosis since 2017, but for patients recruited in 2016 the interviews were done retrospectively over the phone. The definition of delayed diagnosis of CHD used was from a previous study [9]. Delayed diagnosis by doctor was defined from onset of the symptoms to first consultation of a doctor (general practitioner, pediatrician, or other specialist) to definite diagnosis and treatment of CHD, or suspected of CHD. Delay diagnosis by midwifery care was defined as routine for medical consultation and treatment to the midwife, but never suspected of CHD by the midwife. In Indonesia, as in many other low- and middle-income countries, birth assistance providers include highly skilled providers such as doctors and obstetricians, skilled providers such as nurses and midwives, and unskilled providers including traditional birth attendant, relative, others or un-attendant delivery. The delay diagnosis by midwifery care included when the doctor assisted the delivery, but a routine care after birth including immunization was done by the midwives. No trainings for recognizing signs and symptoms of CHD were done previously in the area.

Delay in referral was defined as the time from the doctor (general practitioner, pediatrician, or other specialist) to definite diagnosis, or suspected of CHD to refer to tertiary health care. Delay related to social factors was defined as any personal, cultural and spiritual beliefs that influenced the delayed diagnosis or treatment of CHD after seeking medical attention. Delay related to financial factor was defined as economic factors that influence delayed diagnosis or treatment of CHD after seeking medical attention [9]. We also recorded complications associated with delayed diagnosis at the onset of diagnosis.

Data analysis used STATA version 12.1 (StataCorp, College Station, Texas, USA). Data are presented as means and standard deviations or medians and minimum-maximum or proportions, as appropriate. We did a Kruskal-Wallis test to compare the median of two group. The significance for all categorical variables was assessed using the x^2^ test and *p* < 0.05 was considered to indicate statistical significance.

We did a thematic analysis using the interview data. We first searched for patterns or themes across the different interviews. We then reviewed, defined and named the themes. The themes were related to the reasons of delay diagnosis whether by doctors, midwifery care, delayed in referral, financial or social factor. Finally, reasons associated with delayed diagnosis of CHD were presented thematically based on the top ten common reasons.

The potential factors associated with the delay included epidemiological and clinical variables. The following were evaluated as potential factors associated with the delay: male sex, cyanotic CHD, referred from other hospitals, residence outside the city, non-syndromic, maternal education < 12 years, low family income, normal labour and at term gestation at birth. Univariate analysis was done to determine the significance and strength of the association between each factor and the delay. The significance for all categorical variables were assessed using the X^2^ test and *p* < 0.05 was considered to indicate statistical significance in the univariate analysis. Multivariate logistic regression analysis was conducted to determine the factors independently associated with the delay. All potential factors which were significant in the univariate analysis, were selected and entered into a multivariate analysis and reported as odds ratios and 95% confidence intervals. The Medical and Health Research Ethics Committee of Universitas Gadjah Mada, Indonesia approved this study (KE/FK/0750/EC2020).

## Results

A total of 838 children with CHD were recruited. Table 1 shows the participants’ baseline characteristics. We found delayed diagnosis in CHD was highly prevalent. No antenatal diagnosis was made. Table 2 shows the proportions of delayed diagnosis.

**Table 1:** Baseline characteristics of 838 children with delayed and non-delayed diagnosis congenital heart disease

Characteristics	Total *n* = 838 (%)	Delayed *n* = 510 (%)	No delayed *n* = 328 (%)	*p* value
Age in years, median (min-max)	0.55 (0–17.71)	2.47 (0.04–17.71)	0.13 (0–1.7)	< 0.001
0–1 month	154 (18.4)	10 (2)	144 (43.9)	< 0.001
> 1–12 months	358 (42.7)	174 (34.1)	184 (56.1)	< 0.001
> 12–5 years	133 (15.9)	133 (26.1)	0	< 0.001
> 5–10 years	104 (12.4)	104 (20.4)	0	< 0.001
> 10 – < 18 years	89 (10.6)	89 (17.4)	0	< 0.001
Male sex, n (%)	384 (45.8)	236 (46.3)	148 (45.1)	0.74
Syndrome, n (%)	131 (15.6)	60 (11.8)	71 (21.6)	0.001
Acyanotic CHD, n (%)	679 (81)	373	305	< 0.001
VSD	218 (26)	129 (25.3)	89 (27.1)	0.55
ASD	183 (21.8)	95 (18.6)	88 (26.8)	< 0.001
PDA	177 (21.1)	84 (16.5)	93 (28.3)	< 0.001
PS	60 (7.1)	35 (6.9)	25 (7.6)	0.89
Cyanotic CHD, n (%)	159 (19)	137	23	< 0.001
Pulmonary atresia	44 (5.3)	36 (7)	8 (2.4)	0.003
Tetralogy of Fallot	39 (4.6)	33 (6.5)	6 (1.8)	0.001
DORV	21 (2.5)	19 (3.7)	2 (0.6)	0.004
TGA	15 (1.8)	13 (2.5)	2 (0.6)	0.038
Critical CHD	191 (22.8)	164	27	< 0.001
Source of patients, n (%)				
Community	5 (0.6)	4 (0.8)	1 (0.3)	0.37
Referral status				
Doctor	41 (4.9)	29 (5.7)	12 (3.7)	0.18
Born at Dr. Sardjito Hospital	23 (2.7)	0	23 (7)	< 0.001
1st level of health care	11 (1.3)	7 (1.4)	4 (1.2)	0.74
2nd level of health care	702 (83.8)	446 (87.5)	256 (78)	0.0003
3rd level of health care	45 (5.4)	17 (3.3)	28 (8.5)	0.001
No information	11 (0.1)	7 (0.01)	4 (0.01)	0.85

*CHD* Congenital Heart Disease, *VSD* Ventricular septal defect, *ASD* atrial septal defect, *PDA* patent ductus arteriosus, *PS* pulmonary stenosis, *TOF* tetralogy of Fallot, *DORV* double outlet right ventricle, *TGA* transposition of the great arteries

**Table 2:** Proportion of patients with delayed diagnosis

Delayed Diagnosis	n (%)
All CHD	510/838 (60.8)
Acyanotic CHD	373/679 (54.9)
Cyanotic CHD	137/159 (86.2)
Critical CHD	164/191 (85.9)

*CHD* congenital heart disease

Out of all patients with delayed diagnosis of CHD (*n* = 510), only 382 (74.9%) were successfully interviewed concerning the reasons for delayed diagnosis. Those qualitative data were categorized into several groups as shown in Table 3. The most common reason of delayed diagnosis of CHD was delayed diagnosis by doctor (57.5%).

**Table 3:** Reasons for delayed diagnosis of congenital heart disease

Reasons for delayed diagnosis	*n* = 382 (%)
Delayed diagnosis by doctor	220 (57.5)
Delayed diagnosis related to midwifery care	55 (14.4)
Financial factor	37 (9.7)
Delayed in referral	35 (9.2)
Social factor	35 (9.2)

Our interviews revealed the top ten common reasons of delayed diagnosed reported by the caregivers.

*“My child had recurrent cough and wasted, so the doctor diagnosed tuberculosis infection. After having tuberculosis treatment, he did not improve.”*

*“My child frequently had cough, hard breathing, and did not easily gain weight, but the doctor only suggested to give more feeding.”*

*“My child had been diagnosed CHD since age of two months, but the doctor suggested to wait until he grew older.”*

*“When crying he started to get bluish. The midwife said that it was normal to get bluish when crying.”*

*“I felt it was not OK if my child consumed drugs every day. I decided not seeing the doctor anymore.”*

*“Since birth, my child had been suspected of having CHD, but because of financial problems I saw the doctor just after having health insurance.”*

*“I was sure that it (CHD) could spontaneously close since she looked healthy.”*

*“I did not believe that my child had CHD since she looked OK.”*

*“I never saw the healthcare workers since my child never complained of being sick.”*

*“I decided giving her an alternative medicine rather than seeing the doctor.”*

A total of 9 factors were analysed to estimate the delayed diagnosis in children with congenital heart disease. Univariate analysis identified 8 factors which were significantly associated with the delay including children with cyanotic CHD, referred from other hospitals, residence outside the city, non-syndromic, maternal education ≤ 12 years, low family income, normal labour and at term gestation at birth. By multivariate analysis, children with cyanotic CHD, residence outside the city, non-syndromic, low family income, normal labour and at term gestation were factors that remained independently associated with the delayed diagnosis (Table 4).

**Table 4:** Multivariate analysis of factors associated with delayed diagnosis of congenital heart disease

Factors	Delayed diagnosis *n* = 510	Non-delayed diagnosis *n* = 328	Univariate analysis		Multivariate analysis	
			*p* value	95% CI	*p* value	95% CI
Male sex	236 (46.3)	148 (45.1)	0.670	0.93 (0.71–1.24)		
Cyanotic CHD	137 (26.9)	23 (7.0)	< 0.001	4.87 (3.05–7.76)	< 0.001	4.16 (2.55–6.79)
Referral from other hospital	495 (97.2)	302 (92.1)	0.001	3.04 (1.56–5.92)	0.480	1.32 (0.62–2.81)
Residence outside city	356 (69.8)	165 (50.3)	< 0.001	2.28 (1.71–3.04)	< 0.001	2.08 (1.51–2.85)
Non-syndromic	450 (88.2)	257 (78.4)	< 0.001	2.07 (1.42–3.02)	0.011	1.70 (1.13–2.56)
Maternal education ≤ 12 years	243 (47.6)	118 (36.0)	0.001	1.62 (1.22–2.15)	0.168	1.27 (0.91–1.77)
Low family income	324 (63.5)	166 (50.6)	< 0.001	1.70 (1.28–2.25)	0.043	1.41 (1.01–1.96)
Normal labor	418 (82.0)	200 (61.0)	< 0.001	2.91 (2.12–3.99)	< 0.001	2.43 (1.71–3.44)
At term gestation	469 (92.0)	250 (76.2)	< 0.001	3.57 (2.37–5.37)	< 0.001	3.50 (2.22–5.50)

Children with CHD and delayed diagnosis or late presenters tended to present with several complications (*n* = 615, 73.4%). Table 5 lists the comorbid complications at the onset of diagnosis.

**Table 5:** Complications at the onset of diagnosis among children with CHD

Complications	*n* = 838 (%)
Complications	615 (73.4)
Congestive heart failure	414 (49.4)
Pulmonary hypertension	132 (15.8)
Severe polycythemia	57 (6.8)
Reduced left ventricle function	9 (1)
Infective endocarditis	5 (0.6)
Cerebral abscess	2 (0.2)

## Discussion

The main finding of this study indicated the proportion of delayed diagnosis in children with newly diagnosed CHD reached 6 in 10 children with CHD. A previous study in a low- and middle-income country showed similar results, where delayed diagnosis among children with CHD was 85.1% including 65.3% with acyanotic CHD [11]. These data were much higher than in high-income countries such as Switzerland, with only 10% with CHD considered as delayed diagnosis [11]. However, since the financial cause in our study was only 9.7%, this could not attributed to this high proportion of delayed diagnosis in our study. Other factors such as delayed diagnosis by doctor and midwifery care might be considered as the main reasons for the delayed diagnosis in children with CHD in our population.

In our study, the proportion of delayed diagnosis of CHD was higher in cyanotic than acyanotic CHD. This result is different from previous studies [9, 11]. But, a previous study in a high income country revealed the proportion of delayed diagnosis was 8.9% including in cyanotic CHD of 10.4% and acyanotic CHD of 8.7% [7]. Theoretically, clinical findings in cyanotic CHD should be more obvious than acyanotic CHD because of the bluish discoloration of children with cyanotic CHD due to the right-to-left shunt, which results from deoxygenated blood entering the circulation [12]. This visible condition may encourage parents to seek earlier medical consultation.

One of the reasons why the delayed diagnosis was more common in cyanotic CHD than acyanotic CHD because the appearance of cyanosis depends on the hemoglobin level. In CHD with normal hemoglobin level, cyanosis will appear when the reduced hemoglobin reaches 3 g/dl or about 20% desaturation. In specific conditions such as polycythemia, 15% reduced hemoglobin might cause visible signs of cyanosis. Interestingly, cyanosis does not appear until arterial oxygen saturation is reduced to 50% in children with marked anemia, which may explain why not all children with cyanotic CHD showed cyanotic signs [13].

Cases with critical CHD were 22.8% (191/838) with the proportion of delayed diagnosis of critical CHD of 85.9%, higher than US studies which indicated the prevalence of late detection varies widely (from 7.5 to 62.0%) in critical CHD [8]. The late critical CHD detection was associated with 52% more admissions, 18% more hospitalized days, and 35% higher inpatient costs during infancy [14].

One study in high-income countries reported the decline in late referrals of cyanotic CHD patients is attributable to national neonatal Pulse Oximetry Screening (POS) recommendations [11]. Neonatal POS is a critical factor for screening of CHD in every newborn. Assessment timing ranges at less than 24–48 h of age. Newborns should be referred for cardiology evaluation if oxygen saturation consistently falls below 95% [15]. POS is a moderately sensitive and highly specific test for detection of critical CHD with very low false positive rates [16, 17]. Neonatal POS has not been implemented in routine examination for newborns in Indonesia. In contrast, advanced antenatal screening programs such as fetal echocardiography screening are well-established in high-income countries. This neonatal pulse oximetry screening should be implemented in early detecting critical congenital heart in our setting because of its highly specific test with very low false positive rates [16].

Our findings show diagnosis of CHD in most patients was delayed because of delayed diagnosis by doctor (57.5%), delays related to midwifery care (14.4%), financial factors (9.7%), delays in referral and follow-up (9.2%), and social factors (9.2%). Rashid et al. similarly reported the most patients had delayed diagnosis of CHD because of the delayed first consultation with doctor (37.2%), delayed diagnosis by health professionals (22.5%), delayed referral/follow-up (13.3%), social factors (13%), financial constraints (12.3%), and religious beliefs (1.7%) [9].

Delayed diagnosis by doctor was the most common cause of delayed diagnosis of CHD, defined as the time from first consultation to doctor (general practitioner, pediatrician or other specialist) to definite diagnosis and treatment of CHD or suspected CHD [9]. In primary healthcare settings, lack of awareness about CHD by general practitioners might explain these delays. The clinical features of children with CHD are various, thus the diagnosis is challenging. These clinical signs including cough, dyspnea, and failure to thrive, can be misinterpreted as symptoms of other diseases and managed until the alternate diagnosis of CHD is established. Some patients in our study were diagnosed with tuberculosis and received treatment but they did not get better. Tuberculosis is one of the diseases reported in misdiagnoses of CHD in children due to their similar symptoms namely failure to thrive, and their frequent incidence in Indonesia. These misdiagnoses lead to a lack of follow-up treatment since the physician’s advice to give more feeding will not resolve the main problem.

Additionally, our study revealed 14.4% of patients with delayed diagnosis, routinely received midwifery care. Most deliveries in Indonesia (62.7%) are attended by midwives [2], who are also responsible for children under five and maternal health services in primary healthcare settings, where neonatal CHD screening is not implemented. Our study found the bluish discoloration in children with cyanotic CHD was interpreted as a normal condition. This may be due to the inadequacy of human resources in local maternities center and pediatric services in primary healthcare. Since pediatric cardiac programs and pediatric cardiologists in Indonesia are very few, sometimes pediatricians receive little or no training in pediatric cardiology. This requires training programs to increase knowledge, awareness, referral of CHD among healthcare workers. Further, surgery for CHD must be linked to an early detection and referral system where health workers interact in the diagnosis, management and follow-up of patients [18].

Social factors such as health illiteracy about the disease severity, disease perceptions and stigmatization can slow the advanced treatment. Financial factors such as lack of health insurance complicate this situation. Our study revealed that some caregivers preferred to seek alternative medicine instead of a doctor. This use of traditional remedies is common throughout Indonesia. Understanding Indonesia’s diverse local wisdom is crucial for proper program planning and treatment.

Another barrier that delays CHD diagnosis is delayed referral. Referral in cardiac problems is crucial due to the complications’ unpredictability and their potential to progress rapidly to become severe and life-threatening. Neonatal and child deaths can be prevented if referral systems were structured and in place to allow neonates and children with CHD to reach appropriate health services. Reducing Indonesia’s persistently high neonatal and child mortality rates requires understanding of the neonatal and child referral patterns to inform strategic planning to improve the present referral system.

Children with cyanotic CHD, residence outside the city, low family income, non-syndromic, normal labour and at term gestation at birth were independent factors associated with the delay diagnosis in the multivariate analysis. Timely access to the tertiary hospital for diagnosis of CHD may be hindered when the family income was low and they resided in the rural areas. The delay may be associated with delay in transportation or delay in getting the care in hospitals. Delay in recognition by the parents or health care workers may occur when the children born normally and at term with no obvious genetic syndrome. Many factors including poverty, illiteracy, inadequate health facilities, inappropriately trained health professionals at primary care level, and lack of antenatal care were significantly associated with delayed diagnoses of CHD [9].

Delayed diagnosis of children with CHD significantly affects the outcome. Children tend to present with several complications at the onset of diagnosis. In our study, about half of the children with CHD presented with congestive heart failure. These findings were higher than data reported by Mocumbi et al., reporting only 8.8% of patients with heart failure at the CHD diagnosis [19]. Heart failure is the most common consequence of CHD. A 3/1000/year incidence of heart failure has been reported in children with CHD and has become the main cause of mortality and the second cause of mortality and morbidities in adults with CHD [20–22].

About 15.8% of patients with CHD had pulmonary hypertension, a condition characterized by elevated pulmonary arterial pressure often resulting in right ventricular failure. Pulmonary hypertension was associated with worsening outcome in children with CHD [23]. About 7% of patients had severe polycythemia. Polycythemia may be due to chronic cyanosis which occurs in unoperated tetralogy of Fallot, single ventricle with right ventricular outflow tract obstruction, and Eisenmenger’s syndrome [3].

These complications will potentially increase the morbidities and mortality [3, 24]. Accordingly, the development of early detection or training programs to increase the knowledge in recognizing signs and symptoms of CHD among healthcare staff including doctors and midwives, and better hospital policy and referral system in Indonesia will improve the prognosis and outcomes for children born with CHD. We therefore recommend developing a policy of CHD screening for newborns in Indonesia.

This study has some limitations. The data were collected from a single tertiary hospital, which included only patients treated in this referral facility. The results may not reflect the true proportion of delayed diagnosis of CHD in the community. Another limitation is that only 74.9% of were successfully interviewed regarding delayed diagnosis. This could have affected the study results. Despite these limitations, our study is among the first reports showing the high incidence of delayed CHD diagnosis in Indonesia.

## Conclusions

Our study found six in ten children with CHD had delayed diagnosis. Delayed diagnosis by doctor, delays related to midwifery care, financial factors, delays in referral/follow-up, and social factors were the reasons for the delayed CHD diagnosis. Children with cyanotic CHD, residence outside the city, non-syndromic, low family income, normal labour and at term gestation at birth were independent factors associated with the delay diagnosis.

## Data Availability

The datasets used and/or analysed during the current study available from the corresponding author on reasonable request.

## Abbreviations

CHD: Congenital Heart Disease
VSD: Ventricular septal defect
ASD: Atrial septal defect
PDA: Patent ductus arteriosus
PS: Pulmonary stenosis
TOF: Tetralogy of Fallot
DORV: Double outlet right ventricle
TGA: Transposition of the great arteries
TB: Tuberculosis
POS: Pulse oximetry screening
